# Feasibility of implementing same-day antiretroviral therapy initiation during routine care in Ekurhuleni District, South Africa: Retention and viral load suppression

**DOI:** 10.4102/sajhivmed.v21i1.1085

**Published:** 2020-08-20

**Authors:** Nolundi Mshweshwe-Pakela, Bhakti Hansoti, Tonderai Mabuto, Deanna Kerrigan, Griffiths Kubeka, Elizabeth Hahn, Salome Charalambous, Christopher J. Hoffmann

**Affiliations:** 1 Implementation Research Division, The Aurum Institute, Johannesburg, South Africa; 2 Department of Emergency Medicine, Johns Hopkins School of Medicine, Baltimore, United States of America; 3 Department of International Health, Johns Hopkins Bloomberg School of Public Health, Baltimore, United States of America; 4 School of Public Health, University of the Witwatersrand, Johannesburg, South Africa; 5 Department of Sociology, American University, Washington, United States of America; 6 Department of Medicine, Johns Hopkins University School of Medicine, Baltimore, United States of America; 7 Department of Health, Behavior, and Society, Johns Hopkins Bloomberg School of Public Health, Baltimore, United States of America

**Keywords:** HIV testing, primary care, SDI, ARV initiation, implementation

## Abstract

**Background:**

Same-day initiation (SDI) of antiretroviral therapy (ART) has been advocated as an approach to increase linkage to care and overall ART initiation. Clinical trials have demonstrated impressive benefits. However, questions regarding patient preparedness and retention in care remain for routine implementation of this approach.

**Objectives:**

In this study, we sought to describe SDI of ART during routine care delivery and compare time to ART initiation on longitudinal care outcomes.

**Method:**

We performed a retrospective chart review of 100 consecutive individuals, newly diagnosed with HIV, from 10 health facilities across Ekurhuleni, from January to July 2017. Records were reviewed for a period of 1 year post-diagnosis. Abstracted data included demographics, time to ART initiation, clinic visits and laboratory test results (including viral load testing).

**Results:**

A total of 993 patient records were reviewed, of which 826 were included in the analysis. The majority of patients (752, 91%) had ART initiation recorded, of which 654 (79%) had ART initiated within 30 days, and 224 (27%) had SDI. Uptake of SDI of ART was higher among women (36% vs. 10.4%; *p* < 0.001) and in younger patients (33.7% in those < 29 years; *p* < 0.01). Retention in care at 6 months was achieved in 477 (58%) patients. Of those with 6-month viral loads, 350/430 (73%) had a viral load < 400 c/m. Retention in care and viral suppression were similar among those with SDI of ART and later ART initiation.

**Conclusion:**

Same-day initiation of ART was successfully delivered with similar retention and viral load outcomes as subsequent initiation, providing re-assurance for scale-up of this strategy in routine care.

## Introduction

Timely linkage to antiretroviral therapy (ART) is pivotal to decreasing human immunodeficiency virus (HIV) transmission and reducing HIV-associated morbidity and mortality.^1^ Increasing evidence indicates that same-day initiation (SDI) of ART can increase overall linkage to care (LTC) and the proportion of patients with viral load suppression.^2,3,4,5,6,7^ Randomised studies from South Africa, Haiti and Lesotho have demonstrated the feasibility, acceptability and improved retention over 6 months with SDI of ART when compared with standard practices in research settings.^5,8,9^ In response to clinical trial evidence of the benefits of SDI of ART, the South African Department of Health announced a policy of universal testing and treatment (UTT) in public health facilities in September 2016 and endorsed SDI of ART.^10^ The UTT directorate specified: ‘ART should be started as soon as the patient is ready and within 2 weeks’, with immediate (same-day) initiation prioritised for pregnant or breastfeeding women.^10^ Same-day initiation of ART was further highlighted as a priority in a 2017 South African Department of Health circular.^10,11^ and in the 2017 World Health Organization rapid ART initiation guidelines.^12^

As a counterpoint to SDI of ART enthusiasm, there are concerns regarding the operational feasibility of SDI in public health facilities providing routine care. These concerns include failure to rule-out important opportunistic infections, challenges with assuring adequate renal function for some primary ART regimens and patient readiness to cope with a new HIV diagnosis and initiate life-long ART.^1,13,14,15,16^ Furthermore, SDI of ART may not allow time (and focussed time) to provide important HIV education and adherence counselling that may be valuable for long-term ART success.^16,17,18^ Moreover, a recent South African study demonstrated a reduced rate of retention among same-day initiators.^19^ These reports of early findings warrant further investigation to understand the impact of routine delivery of same-day ART. Lastly, SDI of ART adds a further service burden to an already overwhelmed system. The shortage of ‘nurse-initiation and management of ART’ (NIMART) trained staff, limited resources and long waiting times in facilities pose system-level barriers that may impede the effective implementation of SDI of ART and contribute to the negative outcomes already reported.

In spite of the challenges associated with SDI of ART, it still offers important benefits to both the patient and healthcare system. In addition to early access to a definitive management strategy, SDI obviates the need for repeat visits and is likely a more attractive option for young working adults.^19^ Furthermore, routinisation of SDI may in some way normalise ART initiation, by providing a similar approach to HIV management as other chronic health conditions (i.e. high blood pressure management where antihypertensive medications are often started during the clinic visit without the need for extensive repeat visits or referral to a specialised clinical setting).

This study seeks to describe our experience with SDI across 10 healthcare facilities in the Ekurhuleni District of South Africa and characterise both patients- and health facility-associated factors that impact SDI success, namely retention in care and viral load suppression.

## Materials and methods

### Study design

This is a retrospective clinical review of patients who presented for routine care and tested HIV positive at 10 public sector health facilities in the Ekurhuleni District of South Africa between 01 January 2017 and 31 July 2017. We abstracted clinical data from patient charts (including patient files and HIV testing registers) and electronic databases (Tier.Net^20^ and the South African National Health Laboratory Service [NHLS] database). All data were collected after the pathway of care was completed, there was no interaction with patients and written informed consent was not obtained. All data were de-identified prior to data capture and data analysis.

### Study setting and population

The study took place in the Ekurhuleni District (Gauteng Province) of South Africa. This district has the second-highest district-level HIV prevalence (14.3%) in South Africa and is comprised of urban and peri-urban settings.^21,22^ The study sites consisted of six primary healthcare clinics, three community healthcare centres and one outpatient department at a district-level hospital. Facilities were selected to achieve geographic diversity within the district. All facilities had HIV counselling and testing personnel (lay counsellors) and routinely provide free HIV testing services (HTS) according to the 2015 National HIV Counselling and Testing (HCT) and the UTT policy guidelines.^23,24^

### Sampling and data collection

We aimed to include 100 consecutive adult patients with an HIV-positive diagnosis from each of the 10 health facilities for a total sample of approximately 1000 patients. This sample size was selected to provide an estimate of LTC within 20% of the point estimate, assuming 50% LTC, an alpha of 0.05 and 80% power. Consecutive patients were identified from the HIV testing registers and were included if they were aged 18 or older and had a positive HIV test recorded in the clinic HIV testing register between January 1st and July 31st 2017. Approximately 1 year after the date of diagnosis, paper and electronic clinical records were abstracted for each selected patient, from the clinic at which HIV testing occurred. The data abstracted included sex, age, CD4 count, viral load (HIV RNA), creatinine, ART initiation, HIV care visits, tuberculosis (TB) testing, TB test results and TB preventive therapy prescribing.

### Outcome measures

Linkage to care was defined as documentation of ART prescription. Same-day initiation of ART was defined as having the same date of HIV diagnosis and ART initiation.^25^ Rapid ART initiation that did not occur on the day of HIV testing was defined as initiation between 1 and 7 days after HIV diagnosis.^12^ Retention in care was defined as evidence of a care visit (in the same facility where patients had initiated ART) or HIV-related laboratory testing between 91 and 365 days after ART initiation. Six-month viral suppression was defined as a viral load of < 400 c/mL (for consistency with prior reports) between 4 and 8 months after ART initiation.

### Statistical analyses

Descriptive statistics were used to explore the relationship between patient characteristics and the timing of ART initiation (same-day, 1–7, 8–30, 31–90; > 90 days). Comparisons of factors associated with the timing of ART initiation were conducted using chi-square tests with particular interest in clinic attended, sex, age group, CD4 count and TB testing. Log binomial regression modelling (used to approximate relative risk) was used to assess for associations between the timing of ART initiation and viral suppression and retention in care while adjusting for age, sex, CD4 count and facility. Data were analysed using STATA© v.14 (StataCorp, LLC, Texas).

### Ethical consideration

The study was approved by the University of the Witwatersrand Human Research Ethics Committee, the Johns Hopkins University School of Medicine Institutional Review Board and the Ekurhuleni District Research Committee.

## Results

A total of 993 patient records were identified and abstracted during the study period. Of these, 826 (83.2%) patients were included in the analysis and 167 (16.8%) were excluded. Reasons for exclusion included: 111 (66.5%) had ART recorded prior to testing date, 49 (29.3%) were documented to have transferred out of the healthcare facility and 7 (4.2%) were documented to have died. Of those included, 528 (63.9%) were women, and the median age was 32 years (interquartile range [IQR]; 27, 39). Of the 10 study clinics, the number of patients included in the analysis ranged from 65 to 99 per clinic.

### Patient characteristics at human immunodeficiency virus testing

The majority of patients had CD4 count documentation (610, 73.8%; Table 1) with a median CD4 count of 312 cells/mm^3^ (IQR; 156, 490). Of those with CD4 count documentation, nearly a third had a CD4 count of ≤ 200 cells/mm^3^ (192, 31.5%) (Table 1). Serum creatinine was documented for less than half of the patients (414, 50.1%), of which 14 (3.4%) had an estimated glomerular filtration rate of < 50 ml/min/m^2^. Tuberculosis testing (GeneXpert MTB/Rif) was documented for 45 (5.4%) patients, of which 10 tested positive for TB.

**TABLE 1:** Study population characteristics for 826 patients.

Characteristic	*N*	*%*
Sites (*n*)	10	-
**Age (years)**	-	-
< 29	303	36.7
30–39	335	40.5
≥ 40	188	22.8
**Sex**		
Male	298	36.1
Female	528	63.9
**CD4 count**		
< 200	192	23.2
201–500	278	33.7
≥ 501	140	16.9
Missing	216	26.2

### Timing of antiretroviral therapy initiation

Of the 826 patients included in the analysis, ART initiation within a year of HIV testing was documented for 710 (85.9%; Table 1). Of these, the majority, 654 (92.1%), of patients initiated ART within 30 days after HIV testing (median of 8 days; IQR: 0, 15). A similar proportion of men and women initiated ART (83% and 88%, respectively; *p* = 0.06). Of the 10 patients testing positive for TB, seven were initiated on ART within 30 days with the remaining three initiated on ART within 90 days.

Antiretroviral therapy was initiated on the same day as HIV testing for 221 patients (31.1%). The proportion of patients initiated on ART who had SDI varied considerably by facility, ranging from 8.1% to 61% (10% – 69% for women and 0% – 45% for men). A significantly higher proportion of women, compared with men, initiated ART on the same day (36% vs. 10.4%, *p* < 0.001) (Table 2). Same-day initiation of ART was also more common among those aged < 29 years (102, 33.7%), compared with those aged 30–40 years (89, 26.6%), and those older than 40 years (30, 16%; *p* = 0.009). Lastly, a greater proportion of patients with a higher CD4 count initiated on the same-day: 42 (30%) of those with a CD4 count > 500 cells/mm^3^ compared with 60 (21.6%) among those with a CD4 count between 200 and 500 cells/mm^3^ and 23 (12%) of those with CD4 count < 200 cells/mm^3^ (*p* < 0.001; Table 3). Notably, the CD4 count results were not available to clinicians on the day of HIV testing, so decisions around SDI were not influenced by the CD4 count.

**TABLE 2:** Timing of antiretroviral therapy initiation.

Characteristic	Same-day (*n* = 221)		1–7 days (*n* = 105)		8–30 days (*n* = 328)		31–90 days (*n* = 51)		> 90 days (*n* = 5)		No ART (*n* = 116)		*p*-value (*χ*^2^)
	*n*	%	*n*	%	*n*	%	*n*	%	*n*	%	*n*	%	
**Age (years)**													
< 29	102	33.7	31	10.2	106	35.0	20	6.6	1	0.3	43	14.2	0.009
30–39	89	26.6	46	13.7	134	40.0	16	4.8	2	0.6	48	14.3	
≥ 40	30	16.0	28	14.9	88	46.8	15	8.0	2	1.1	25	13.3	
**Sex**													
Male	31	10.4	49	16.4	141	47.3	24	8.1	2	0.7	51	17.1	< 0.001
Female	190	36.0	56	10.6	187	35.4	27	5.1	3	0.6	65	12.3	
**CD4 count**													
< 200	23	12.0	34	17.7	105	54.7	19	9.9	1	0.5	10	5.2	< 0.001
201–500	60	21.6	44	15.8	131	47.1	23	8.3	2	0.7	18	6.5	
> 501	42	30.0	19	13.6	63	45.0	6	4.3	2	1.4	8	5.7	
Missing	96	44.4	8	3.7	29	13.4	3	1.4	0	0.0	80	37.0	

ART, antiretroviral therapy.

**TABLE 3:** Viral suppression and retention in care at 6 months for those who are on antiretroviral therapy.

Characteristic	Viral suppression (< 400) at 6 months (*n* = 455)					Retained in care at 6 months (*n* = 710)				
	Suppressed		Not suppressed		*p*-value (Chi-squared)	Retained		Not retained		*p*-value (Chi-squared)
	*n*	%	*n*	%		*n*	%	*n*	%	
**Age (years)**										
< 29	131	80.0	33	20.1	0.31	171	65.8	89	34.2	0.36
30–39	136	75.6	44	24.4		189	65.8	98	34.2	-
≥ 40	92	82.9	19	17.1		117	71.8	46	28.2	-
**Sex**										
Male	123	77.4	36	22.6	0.55	169	68.4	78	31.6	0.61
Female	236	79.7	60	20.3		308	66.5	155	33.5	-
**CD4 count**										
< 200	93	75.0	31	25.0	0.16	128	70.3	54	29.7	0.45
201–500	124	81.6	28	18.4		168	64.6	92	35.4	-
≥ 501	74	85.1	13	14.9		93	70.4	39	29.6	-
No value	68	73.9	24	26.1		88	64.7	48	35.3	-
**Time to ART**										
Same-day	118	78.1	33	21.8	0.50	153	69.2	68	30.8	0.47
1–7 days	56	83.6	11	16.4		76	72.4	29	27.6	-
8–30 days	160	79.2	42	20.8		210	64.0	118	36.0	-
31–90 days	23	74.2	8	25.8		34	66.7	17	33.3	-
> 90 days	2	50.0	2	50.0		4	80.0	1	20.0	-

ART, antiretroviral therapy.

### Retention in care by timing of antiretroviral therapy initiation

A total of 477 patients (67.2% of patients who initiated ART) met our definition of retention in care at 6 months. There was no association between retention in care and time of ART initiation; 69.2% (153) of those who initiated same-day ART were retained at 6 months compared with 66.1% (286) who initiated ART between 1 and 30 days post-diagnosis (*p* = 0.47; Table 3). In a model-adjusted for sex, age and CD4 count, time of ART initiation did not significantly impact the relative likelihood retention in care at 6 months (Table 4).

**TABLE 4:** Log-binomial logistic regression adjusted for age, gender, CD4 count and antiretroviral therapy initiation group.

Characteristic	Viral suppression at 6 months			Retention in care at 6 months		
	Risk ratio		*p*-value	Risk ratio		*p*-value
	*n*	95% CI		*n*	95% CI	
**Age (years)**						
< 29	Ref	-	-	Ref	-	-
30–39	0.93	0.49–1.78	0.84	1.19	0.78–1.82	0.41
≥ 40	1.64	0.74–3.61	0.22	1.50	0.90–2.48	0.12
**Sex**						
Male	Ref	-	-	Ref	-	-
Female	1.46	0.80–2.64	0.21	0.93	0.63–1.40	0.75
**CD4 count**						
< 200	Ref	-	-	Ref	-	-
201–500	1.58	0.85–2.92	0.15	0.82	0.53–1.25	0.35
≥ 501	1.90	0.88–4.06	0.10	1.05	0.63–1.77	0.84
**Time to ART initiation**						
Same-day	Ref	-	-	Ref	-	-
1–7 days	1.92	0.77–4.90	0.16	0.93	0.51–1.71	0.82
8–30 days	1.34	0.67–2.65	0.41	0.73	0.45–1.17	0.19
31–90 days	1.04	0.37–2.96	0.93	0.79	0.38–1.64	0.52
> 90 days	0.26	0.03–2.17	0.21	1.44	0.15–13.56	0.75

ART, antiretroviral therapy.

### Viral load suppression by timing of antiretroviral therapy initiation

A total of 455 patients (64.1% of patients who initiated ART) had a 6-month viral load result documented, of which 359 (79%) were virally suppressed (viral load < 400 c/mL; Table 3). There was no significant association between viral suppression and time to ART initiation; 78.1% (118) of patients who initiated same-day ART were virally suppressed compared with 80.3% (216) patients who initiated ART between 1 and 30 days post-diagnosis (*p* = 0.5; Table 4). In a model adjusted for sex, age group and CD4 count, time of ART initiation did not significantly impact the relative likelihood of viral suppression (Table 4).

## Discussion

We found that just over a quarter of patients at 10 routine care facilities in South Africa initiated same-day ART. Of those initiating ART, 58% were retained in care at the same clinic at 6 months, and 58% of those retained in care at 6 months had a viral load of < 400 c/mL (80% of those with viral load test results). There was no significant association between the timing of ART initiation and viral suppression or retention in care. Furthermore, we identified that same-day ART achieves similar retention in care and viral suppression when compared with later initiation. These findings suggest that provision of SDI does not adversely impact long-term care outcomes. It is plausible that the overall retention and viral load suppression is a superior strategy to delayed ART initiation, because of less time lost from care, by reducing the time between the testing visit and subsequent clinic visit. These are important and encouraging early findings from the routine implementation of SDI that help to expand the understanding of the use of SDI beyond clinical trial settings.

We found several patient-level characteristics that impacted the likelihood of SDI, namely sex, age and CD4 count. Healthier, young women were more likely to be initiated on ART and initiated on the same day. It is unclear if this was because of provider biases in terms of who was offered SDI of ART, or a potential pitfall when engaging older male patients into SDI of ART. It is possible that higher rates of ART initiation in women are accounted for by same-day ART among pregnant women as part of prevention of mother-to-child transmission (PMTCT); the overall lower engagement in care among men may also have contributed to our findings.^26,27,28^ In contrast to general ART initiation studies and an SDI study from Zimbabwe, we observed that younger patients (men and women) were more likely to have SDI compared with older patients.^29^ This is an intriguing finding and contrasts with findings that older individuals are more likely to initiate ART.^30,31^ It is plausible that SDI may have an especially important role in engaging young adult patients in care.^30^ Overall, the most substantial variation in SDI was by facility (10 – 69% for women and 0 – 45% for men). This variability was not associated with the type of health facility or structure, nor daily headcount, suggesting that health facility practice and provider discretion were the primary determinants of whether or not a patient received SDI. Further qualitative research within healthcare facilities to understand facility-based barriers to SDI is needed to support this initiative.

Studies of retention in care with SDI are limited to studies of PMTCT and research studies.^32^ Research studies on SDI have reported increased retention in care, whereas some PMTCT studies have reported a higher loss from care with same-day ART.^5,6,32,33,34,35,36,37^ Same-day ART may improve outcomes by reducing the cost of care and enhancing the perceived value of ART because of the immediacy of receiving the therapy.^38,39^ The findings of our study present a stark contrast to a recently published study led by Lilian et al., which presents data on the national impact of SDI on retention in care.^19^ Routinely collected data from TIER.Net (*n* = 32 290) were analysed for HIV-infected adults who were newly initiating ART in Johannesburg or Mopani districts between October 2017 and June 2018. Their study identified a significant increase in lost to follow-up (LTFU) at 30.1% in the SDI group compared with 22.4%, 19.8% and 21.9% among clients initiating ART 1–7 days, 8–21 days and ≥ 22 days after HIV diagnosis, respectively.^19^ Although LTFU was similar to our study (overall LTFU was 32.8%, 233/710), the association between the timing of ART initiation and LTFU was not found. Although our study was smaller, it benefitted from the triangulation of multiple data sources that may capture individuals missed in the Tier.Net system. This likely reveals the bias of using a single data source, which may underestimate LTC. Additionally, it is also possible that there is a heterogeneity in the success of SDI by district and even by clinic. Our study within a single district (Ekurhuleni) had more promising LTC results than those in the neighbouring districts of Johannesburg and Mopani. A deeper dive into the implementation practices across districts and their relative LTC outcomes is required to understand this phenomenon further.

This study has the strength of assessing multiple clinics that provided routine care in a real-world setting without augmentation from research or academic staff. We also used multiple sources for data collection, paper and electronic and both local and national, to the covert possibility of missing or incorrect data in one source. Some notable limitations arise from the use of chart review to assess successful LTC. Linkage to care and ART initiation required documentation in a file at the same clinic as HIV testing occurred. Patients who initiated ART at other facilities were considered as not having initiated ART. Furthermore, we also did not abstract a reason for visit at the time of HIV testing, making it impossible to compare ART for PMTCT with other initiation reasons. Finally, time to ART initiation was not randomised. As a result, more motivated individuals may have started on the same day. It is also plausible that some same-day initiators were just doing what they were told and were not motivated. Comparing early ART (1–7 days after diagnosis) with same-day ART provides a group with motivation demonstrated by a prompt return to the clinic to the potentially more heterogeneous same-day group. However, the similar outcomes between these two groups are reassuring.

## Conclusion

This study demonstrates the feasibility of SDI of ART and similar outcomes with subsequent initiation. These findings are reassuring and should support continued scale-up of SDI of ART. The finding of higher SDI of ART among younger individuals may suggest that this approach is especially promising to improve care engagement among this population. Operational research should be continued to identify challenges and barriers to inform programmatic delivery of same-day ART.
